# Preconcentration of Trace Amounts of Pb(II) Ions without Any Chelating Agent by Using Magnetic Iron Oxide Nanoparticles prior to ETAAS Determination

**DOI:** 10.1100/2012/640437

**Published:** 2012-04-20

**Authors:** S. Z. Mohammadi, T. Shamspur, M. A. Karimi, E. Naroui

**Affiliations:** ^1^Department of Chemistry, Payame Noor University, P.O. Box 19395-4697, Tehran, Iran; ^2^Department of Chemistry, Payame Noor University, P.O. Box 76175-351, Kerman, Iran; ^3^Department of Chemistry, Shahid Bahonar University, P.O. Box 76175-133, Kerman, Iran; ^4^Department of Chemistry & Nanoscience and Nanotechnology Research Laboratory (NNRL), Faculty of Sciences, Payame Noor University, P.O. Box 78168-5833, Sirjan, Iran; ^5^Department of Chemistry, Sistan & Baluchestan University, P.O. Box 98167-45639, Zahedan, Iran

## Abstract

This work investigates the potential of magnetic Fe_3_O_4_ nanoparticles as an adsorbent for separation and preconcentration of trace amounts of lead from water samples prior to electrothermal atomic absorption spectrometry (ETAAS) determination. No chemical modifier is required in graphite furnace. Pb(II) ion was adsorbed on magnetic Fe_3_O_4_ nanoparticles in the pH range of 5.5–6.5, and then magnetic nanoparticles (MNPs) were easily separated from the aqueous solution by applying an external magnetic field; so, no filtration or centrifugation was necessary. After extraction and collection of MNPs, the analyte ions were eluted using HNO_3_ 1.0 mol L^−1^. Several factors that may affect the preconcentration and extraction process, such as pH, type, and volume of eluent, amount of MNPs, sample volume, salting out effect, and interference ions were studied and optimized. Under the best experimental conditions, linearity was maintained between 0.005–0.5 ng mL^−1^. Detection limits for lead were 0.8 ng L^−1^ based on 3S_b_. The relative standard deviation of seven replicate measurements of 0.05 ng mL^−1^ of Pb(II) ions was 3.8%. Finally, the method was successfully applied to extraction and determination of lead ions in the water and standard samples.

## 1. Introduction

In recent years, water pollution caused by heavy metals is one of the major economic and environmental problems all over the world. Among heavy metal ions, Pb(II) is considered to be a non-biodegrade-like organic pollutant in water and attracted more attention due to its toxicity, persistent in nature particularly, even at low concentrations [1–3]. Consequently, the development of reliable methods for the removal and determination of lead in environmental and biological samples is of particular significance [4].

Several techniques have been used to determinate the levels of lead in environmental and biological samples. These include electrothermal atomic absorption spectrometry (ETAAS) [5], inductively coupled plasma mass spectroscopy [6], inductively coupled plasma optical emission spectroscopy (ICP-OES) [7], and flame atomic absorption spectroscopy (FAAS) [8]. However, their sensitivity and selectivity are usually insufficient for direct determination of the Pb(II) ions at a very low concentration level in complex sample matrices [9]. In order to overcome these problems, separation and preconcentration of Pb(II) ions become necessary, particularly when it exists at trace levels of concentration. A number of separation/preconcentration procedures have been used for trace metal determinations: these include precipitation/coprecipitation [10], liquid-liquid extraction [11], and solid phase extraction (SPE) [12].

Nowadays, SPE is a well-established technique and has been applied for the preconcentration and cleaning-up of numerous different classes of compounds in a variety of matrices by virtue of its high enrichment factor, high recovery, rapid phase separation, low consumption of organic solvents, and compatibility with different detection techniques [13–19]. In some cases, however, due to the limited rate of diffusion and mass transfer, extraction time of ordinary SPE processes is usually long [20], it is particularly evident when extracting very low amount of the target analytes from large volumes of samples. Thus, novel SPE modes that can facilitate mass transfer are highly desirable.

It is commonly acknowledged that the sorbent plays a very important role in the SPE technique, which is related to the analytical sensitivity, precision, and selectivity. Recently, various types of solid-phase sorbent have been developed [21]. Magnetic nanoparticles such as magnetite (Fe_3_O_4_) and maghemite (*γ*-Fe_2_O_3_) can be used as novel and excellent adsorbents due to their unique advantages over traditional microsized adsorbents [22]; they possess not only high surface area which can exhibit higher adsorption capacity for analytes, but also strong superparamagnetic properties which can meet the need of rapid extraction of large volume samples by employing a strong external magnetic field. In recent years, MIONs have been applied for the separation of trace organic compounds and metal ions in various samples [23–29].

To the best of our knowledge, there has been no study conducted on the use of magnetic iron oxide nanoparticles (MIONs) for the separation and preconcentration of trace metals without addition of chelating agent and without any modification of MIONs. Therefore, the main objective of this study is to investigate the preconcentration of Pb(II) on MIONs, prior to ETAAS determination in water and certified environmental samples.

## 2. Experimental

### 2.1. Reagents and Solutions

All chemicals were of analytical-reagent grade, and all solutions were prepared with deionized water. The laboratory glassware was kept overnight in a 1.4 mol L^−1^ HNO_3_ solution. Before using, all the glassware was washed with deionized water and dried. Stock solution of lead at a concentration of 1000.0 *μ*g mL^−1^ was prepared from Merck (Darmstadt, Germany). Working reference solutions were obtained daily by stepwise dilution from stock solution. A solution of 10% (w/v) NaCl (Merck) was prepared by dissolving of 10 g of NaCl in 100 mL of de-ionized water. Buffer solution was prepared from 0.1 mol L^−1^ sodium dihydrogen phosphate and 0.1 mol L^−1^ disodium hydrogen phosphate for pH 6. The solution of alkali metal salts (1% w/v) and various metal salts (0.1% w/v) was used to study the interference ions.

### 2.2. Instrumentation

The measurements for lead determination were performed with a Shimadzu AA-680G atomic absorption spectrometer equipped with GFA-4A graphite furnace and deuterium background corrector. Lead hollow-cathode lamp was used for absorbance measurements at wavelength of 283.3 nm and operated at 7.0 mA, with a spectral bandwidth of 0.3 nm. Peak area absorbance values were measured. Pyrolytically coated graphite tubes (Schunk, Germany) with a preinstalled pyrolytic graphite L'vov platform were used. Argon was used as sheathing gas; the internal gas flow in the graphite tube was interrupted during the atomization step. The instrumental parameters for ETAAS determination of lead are given in Table 1. A Metrohm 692 pH (Herisau, Switzerland) was used for pH measurements.

### 2.3. Preparation of MIONs

The MIONs were synthesized by coprecipitation of a stoichiometric mixture of ferrous and ferric chlorides (molar ratio 1 : 2) in an ammonium hydroxide solution with constant stirring [30]. The nanoparticles were collected by the magnet and thoroughly washed with deionized water to remove excess amounts of ammonium hydroxide.

### 2.4. Characterization of MIONPs

The microstructure of the MIONs was observed by transmission electron micrograph (TEM) image and showed that the adsorbent had a regular surface with an average size less than 50 nm.

Relative magnetization curve was determined at room temperature using a Quantum Design MPMS 5 superconducting quantum interface device magnetometer. The magnetic MIONs were characterized by a high magnetic moment when placed under a high magnetic field. The magnetic moment in the absence of an applied field was subtracted from the result. The magnetization curve exhibited zero magnetization upon the removal of magnetic field, which is a characteristic behavior of superparamagnetic particles. Magnetic MIONs did not retain any magnetization after the removal of an external magnetic field which proved the superparamagnetic characteristic of these nanoparticles.

Another important parameter for practical applications of synthesized Fe_3_O_4_ is their magnetization. Due to the asymptotic increase of magnetization for high fields, the saturation magnetization value can be obtained from the fitting of the M versus 1/H curves, extrapolating the magnetization value of 1/H to 0 [31]. The results were shown that the saturation magnetization for uncoated-NPs is 55.7 emu g^−1^, which is lower than that of bulk magnetite (92 emu g^−1^) [32]. This reduction might suggest a mixture with the maghemite phase.

### 2.5. General Procedure

The extraction procedure was carried out in a batch process mode. Fifty mL of each standard and sample was placed in a beaker. To each beaker, 2 mL of 0.1 mol L^−1^ phosphate buffer (pH 6), 1 mL of 10% (w/v) NaCl, and 100 mg MIONs were added. Then, beakers were stirred for 5 min. The beaker was placed on the magnet, and nanoparticles were collected. After decanting the supernatant solution, the collected MIONs were washed with 1.0 mL of 1.0 mol L^−1^ HNO_3_ solution in order to desorb the adsorbed ions. Then, analyte ions in the eluent were determined by ETAAS.

### 2.6. Sample Preparation

Tap, river, mineral, and seawater samples were collected in acid-leached polyethylene bottles. The only pretreatment was acidification to pH 2 with nitric acid, which was performed immediately after collection, in order to prevent adsorption of the metal ions on the flask walls. The samples were filtered before analyses through a cellulose membrane (Millipore, Bedford, MA) of 0.45 *μ*m pore size. Ten milliliter of each water sample was transfered to calibrated flask and was made to 250.0 mL with deionized water in a calibrated flask.

Twenty milliliter of urine samples was given and heated for 1 h after addition of 15 mL concentrated HNO_3_ and 4 mL HClO_4_ 70%. The content of the flasks was diluted with deionized water and filtered through a Whatman no. 40 filter paper into a 100 mL calibrated flask, and its pH was adjusted to 6 [18].

## 3. Results and Discussion

In this study, a combination of SPE and ETAAS was developed for determination of trace amounts of lead. Several factors that may affect the preconcentration and extraction process, including pH, type, and volume of eluent, sample volume and matrix effect were optimized. The optimizations were carried out on 50 mL of aqueous solution containing 2.5 ng of lead ions.

### 3.1. Effect of pH

**Figure 1:**
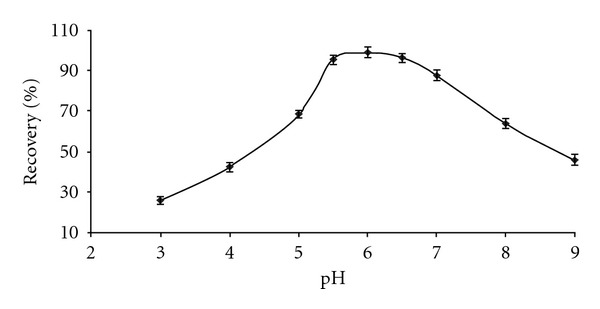
Effect of pH on the recovery of lead. Conditions: Pb(II), 2.5 ng; buffer, 2 mL; NaCl 10% (w/v), 1 mL; MIONs, 100 mg; sample volume, 50 mL.

Since the pH of the aqueous sample solutions is an important analytical factor in the SPE studies of metal ions, the influence of pH on the preconcentration of lead ions was examined in the pH range of 3–9, keeping the other parameters constant. It was found that the lead was quantitatively adsorbed on the sorbent in the pH range 5.5–6.5. The pH curves for adsorption of Pb(II) ions are shown in Figure 1. In subsequent studies, the pH was maintained at approximately 6. Addition of 1–6 mL of buffer did not have any effect on the adsorption. Therefore, 2 mL of 0.1 mol L^−1^ phosphate buffer solution was used in all subsequent experiments. 

### 3.2. Effect of Contact Time

Effects of contact time on the adsorption of Pb(II) by MIONPs were studied in the range of 1 to 30 min. The results showed that the recovery percent increased sharply to 4 min and remained constant. Therefore, 5 min was used in all subsequent experiments.

### 3.3. Effect of the Adsorbent Amount

The required amount of MIONs (5–200 mg) for the complete adsorption of the lead ions in 50 mL solution containing 2.5 ng of lead ions was also studied. The results showed that the recovery percent increased to 10 mg and remained constant. Therefore, 100 mg of MIONPs was used in all subsequent experiments.

### 3.4. Effect of Salt

Sodium chloride was used to investigate the influence of ionic strength on the extraction efficiency. For investigating the influence of the ionic strength on the extraction of Pb(II) ions, several experiments were performed by adding varying volumes of NaCl 10% from 0.0 to 1.5 mL. The rest of the experimental conditions were kept constant. The results were showed that the extraction efficiency was increased to 0.75 mL and then remained constant in the range of 0.75 to 1.5 mL. Therefore, 1 mL NaCl 10% was used in all further experiments.

### 3.5. Elution of the Adsorbed Ions

Another important factor which affects the preconcentration procedure is the type, volume, and concentration of the eluent used for the removal of the analyte ions from the sorbent. Optimization of the elution conditions was performed in order to obtain the maximum recovery with the minimal concentration and volume of the eluent. For this purpose, HNO_3_, HCl, KSCN, and Na_2_S_2_O_3_ (1 mL of 1.0 mol L^−1^) were used as eluent solution. The extraction efficiency for HNO_3_, HCl, KSCN, and Na_2_S_2_O_3_ as an eluent solutions was 98.7 and 93.4; 84.9 and 91.5, respectively. Therefore, 1.0 mL of 1 mol L^−1^ HNO_3_ was used in all subsequent experiments.

### 3.6. Effect of the Sample Volume

The volume of an aqueous solution containing 1.0 ng of Pb(II) ions was varied in the range of 10–250 mL in steps under the optimum conditions. It was observed that absorbances were almost constant up to 200 mL of the aqueous phase. With respect to eluent volume (1.0 mL), preconcentration factor (the ratio of the highest sample volume to the lowest eluent volume) for the analyte ions was obtained 200. However, for convenience, all the experiments were carried out with 50 mL of the aqueous phase.

### 3.7. Effect of Interference

Various salts and metal ions were added individually to a solution containing 2.5 ng of Pb(II) ions, and the general procedure was applied. The tolerance limit was set as the concentration of the diverse ion required to cause ±5% error. The results obtained are given in Table 2. Most of the ions examined did not interfer. Thus, the proposed method is selective and can be used for determination of Pb(II) ions in complex samples without any prior separation.

### 3.8. Adsorption Capacity

To determine adsorption capacity of MIONs, 50 mL of aqueous solution containing 3.0 mg of Pb(II) ion at pH 6 was added to 100 mg of MIONs. After shaking for 30 min, the MIONs were separated and the supernatant solution was determined by FAAS. The capacity of MIONs for Pb(II) ions was found to be 28.6 mg g^−1^.

### 3.9. Analytical Figures of Merit

Under the optimized conditions, calibration curves were constructed for the determination lead according to the general procedure. Linearity in the original solution was maintained between 0.005–0.5 ng mL^−1^ with a correlation coefficient of 0.9985 (*A* = 1.481*C* + 0.002, where *A* is the absorbance value of the eluent and *C* is the concentration of Pb (ng mL^−1^)). Seven replicate determinations of 0.05 ng mL^−1^ of Pb(II) ions in the original solution gave a mean absorbance of 0.078 with a relative standard deviation 3.8%. The detection limit was determined as three times the standard deviation (7 replicate measurements) of the absorbance of a blank sample. The detection limit for lead in the original solution was 0.8 ng L^−1^.

### 3.10. Accuracy of the Method

The accuracy and applicability of the proposed method has been applied to the determination of Pb(II) ions in NIST CRM 1643e (National Institute of Standard and Technology, Trace elements in water). The amount of lead in NIST CRM 1643e was found to be 19.47 ± 0.26 ng mL^−1^. It was found that there is no significant difference between the result obtained by the general procedure and the certified result (19.63 ± 0.21). The *t*-test was applied to both sets of results and showed that there was no significant difference at the 95% confidence level.

### 3.11. Application to Real Samples

The general procedure has been applied to the determination of Pb(II) ion content in tap water, seawater, mineral water, and urine samples by using 50.0 mL of each sample. The results are given in Table 3. Also, the recovery of Pb(II) ions from samples spiked with known amounts of lead ions was studied. The results are shown in Table 3. As can be seen from the results in Table 3, the added lead ions were quantitatively recovered from the water samples by the general procedure. These results demonstrate the applicability of the procedure for lead determination in water samples.

### 3.12. Comparison

A comparison between the proposed method and the other reported preconcentration methods [5, 33–36] for the Pb(II) ion extraction from water samples is given in Table 4. The obtained detection limits by the proposed method are comparable to most of those reported in the literature.

## 4. Conclusions

It can be concluded from the results that MIONs are an effective sorbent for separation and preconcentration of trace amounts of lead from various water samples. The greatest advantage of this method is that desired materials are separated from solution by a simple and compact process while less secondary wastes are produced. Other advantages are avoidance of channeling effects that are common in packed beds; simple, rapid, reproducible, and low analysis cost. Also, MIONs did not retain any magnetization after the removal of an external magnetic field which proved the superparamagnetic characteristic of these nanoparticles.

## Figures and Tables

**Table 1:** Operating parameters for ETAAS determination of lead.

Heating step	Temperature (°C)	Ramp (°C min^−1^)	Hold (s)	Argon flow rate (mL min^−1^)
Drying	120	10	20	250
Pyrolysis	400	10	40	250
Atomization	1200	0	3	0
Cleaning	2000	1	2	250

**Table 2:** Tolerance limit of the foreign ions.

Foreign ions	Interference/Pb(II) ratio	Recovery (%)
H_2_PO_4_^−^, HPO_4_^2−^	7000	95
Na^+^, K^+^	5000	95
Ca^2+^, Mg^2+^	3000	105
Co^2+^	800	95
Cu^2+^, Mn^2+^	1000	105
Fe^2+^, Fe^3+^	400	95
Ni^2+^, Zn^2+^	1000	105
Al^3+^	100	95
Cr^3+^	800	95
Sn^2+^, Cd^2+^	600	105
Ag^+^	200	95
Sb^3+^, Cd^2+^	500	96

Conditions were the same as Figure 1.

**Table 3:** Determination of lead in real samples.

Sample	Lead amount (ng mL^−1^)		Recovery (%)
	Added	Found*	
Tap water	0.0 5.0	5.6 ± 0.3 10.5 ± 0.6	— 98.0
Mineral water 1	0.0 5.0	6.9 ± 0.4 12.1 ± 0.7	— 104.0
Seawater	0.0 5.0	4.7 ± 0.3 9.8 ± 0.7	— 102.0
River water, Kohpayeh, Kerman	0.0 5.0	2.9 ± 0.2 7.7 ± 0.7	— 96.0
Urine	0.0 1.0	0.073 ± 0.004 1.085 ± 0.068	— 101.2

*Average ± standard deviation (*n* = 3).

**Table 4:** Comparison of the proposed methods with the other method.

Enrichment method	Detection method	Sample volume (mL)	Detection limit (ng mL^−1^)	Reference
SPE	ETAAS	100	0.11	[5]
HF-LLSMET*	ETAAS	20	7.0	[33]
—	ETAAS	2 g blood	1.77	[34]
Slurry	ETAAS	0.02	0.4	[35]
SPE	ICP-OES	3	1.13	[36]
SPE	ETAAS	50	0.8	This work

*Hollow-fiber liquid-liquid-solid microextraction technique.
